# Cluster Analysis of Physical and Cognitive Ageing Patterns in Older People from Shanghai

**DOI:** 10.3390/diagnostics6010011

**Published:** 2016-02-22

**Authors:** Stephan Bandelow, Xin Xu, Shifu Xiao, Eef Hogervorst

**Affiliations:** 1 School of Sports, Exercise and Health Sciences, Loughborough University, Ashby Road, Loughborough, LE11 3TU, UK; 2 Memory, Ageing and Cognition Centre, National University Health System, Singapore 117600, Singapore; phcxx@nus.edu.sg; 3 Department of Geriatric Psychiatry, Shanghai Mental Health Centre, School of Medicine Shanghai Jiaotong University, Shanghai 200030, China

**Keywords:** aging, frailty, memory, gait, grip strength, balance, cognition

## Abstract

This study investigated the relationship between education, cognitive and physical function in older age, and their respective impacts on activities of daily living (ADL). Data on 148 older participants from a community-based sample recruited in Shanghai, China, included the following measures: age, education, ADL, grip strength, balance, gait speed, global cognition and verbal memory. The majority of participants in the present cohort were cognitively and physically healthy and reported no problems with ADL. Twenty-eight percent of participants needed help with ADL, with the majority of this group being over 80 years of age. Significant predictors of reductions in functional independence included age, balance, global cognitive function (MMSE) and the gait measures. Cluster analysis revealed a protective effect of education on cognitive function that did not appear to extend to physical function. Consistency of such phenotypes of ageing clusters in other cohort studies may provide helpful models for dementia and frailty prevention measures.

## 1. Introduction

An elusive and controversial concept, frailty is thought to be highly prevalent in old age, particularly in those with low education and low socioeconomic status [1]. The majority of published diagnostic criteria for frailty focus on multiple tests of physical function, for example, the widely used “Fried criteria” [1]. According to Fried (2001), the phenotype of clinical frailty is characterized by a critical mass of three or more “core frail elements” which are: (i) weight loss >10 lbs. in past year; (ii) weak grip strength (lowest quintile); (iii) exhaustion (by self-report); (iv) slow gait speed (lowest quintile); and (v) low physical activity (lowest quintile). Similarly, Ensrud (2007) identified the frailty phenotype as having the following components: (i) unintentional weight loss; (ii) self-reported fatigue; and (iii) diminished physical activity, defined as impaired grip strength and reduced gait speed [2]. Campbell (1997) [3] measured frailty by using the following specific tests: (i) grip strength; (ii) chair stand; (iii) sub-maximal treadmill performance; (iv) 6 min walking test; (v) the Static Balance Test; (vi) Body Mass index (BMI); (vii) arm muscle area (to assess sarcopenia: the muscle loss associated with frailty); and (viii) the Mini Mental State Examination (MMSE) [4] to assess cognitive impairment. Cognitive decline may lead to increased risk of physical frailty, but frailty can also precede possible later dementia. One study showed that at post-mortem, Alzheimer’s disease (AD) pathology was associated with frailty in both people with and without dementia [5]. Risk for frailty was doubled in people with AD pathology, independent of a history of other disease and level of physical activity. Even those who were physically frail with no cognitive impairment at baseline had a higher risk of developing AD at follow-up. Frailty may thus also be an early predictor of AD risk, occurring before memory loss.

However, the fact that only the criteria developed by Campbell and colleagues [3] include a measure of cognitive function is indicative of the uncertainty surrounding the relationship between physical frailty and cognitive decline. Physical, psychological, cognitive and social factors may all contribute to this syndrome and may need to be taken into account in its definition and treatment [6,7]. Including tests of psychological and cognitive status allows for identification of specific areas of potential disability, which could perhaps be targeted by specific interventions. It is often assumed that physical and cognitive/psychological decline are linked, but there are few epidemiological studies to substantiate this. If physical and cognitive abilities are linked, physical interventions may be effective in reducing cognitive decline. However, physical exercise intervention studies in the elderly have not found consistent positive effects on cognitive function [8,9].

Whether only one or several assessments are useful in diagnoses of frailty is also important for the complexity and cost of screening. In contrast to criteria requiring multiple and costly assessments, Abate (2007) suggested that both self-report and an objective evaluation of physical performance would be the best indicators of frailty in elderly subjects [7]. Others also used fewer tests than those described in the criteria above. For instance, Ravaglia (2008) only used a cut-off point of 24 on the Tinetti gait and balance performance test to obtain a “frailty score” [10]. However, their prognostic score was not adequately tested in a cohort of elderly, and sensitivity and specificity of this test needed further investigation. Syddall (2003) only examined grip strength as a single marker for frailty [11]. Guyatt (1985) investigated participants’ performance on a 6-min walking test and concluded that this by itself was a useful and acceptable measure of functional exercise capacity, and a meaningful predictor of frailty [12].

The present study employed widely used tests for frailty and dementia to assess whether physical and cognitive impairments affecting activities of daily living would cluster together in the elderly. Cognitive function measures included the Mini-Mental State Examination (MMSE) [4], which is widely used in dementia diagnosis. The Hopkins Verbal Learning Test [13,14] is less well known, but was earlier described for its validity in diagnosing dementia in Chinese elderly from Beijing and Shanghai, using the same cut-off scores as those used in Australia, Indonesia and Oxfordshire (UK) [13,14,15]. Activities of Daily living (ADL) were also assessed as they are highly relevant to quality of life and are widely used in dementia, frailty and disability diagnosis.

Tests for physical frailty, such as grip strength, gait speed and balance, were identified based on criteria mentioned above. Loss of grip strength is strongly associated with increasing chronological age [16]. Lower grip strength is associated with incident as well as prevalent disability, and can be predictive of morbidity and mortality [17]. Rantanen (1999) also found that hand grip strength was highly predictive of functional limitations and disability 25 years later [17]. The Timed Get Up and Go Test (TUG) was examined in a community-based study in America and proved to be a good predictor of falls (sensitivity 87%, specificity 87%) [18]. A community-based study conducted among elderly in Ireland concluded that lower score on the TUG test was associated with lower level of cognitive performance, including executive function, attention and memory [19]. The results indicated that the TUG can be used to predict risk of being frail and cognitively impaired. A loss of balance can increase the risk for falls, which in turn can increase dependency, dementia risk and morbidity [8]. The Berg balance test [20] was also used in this study. It was developed as a clinical performance-oriented measure of functional balance specifically in elderly. It was found to be strongly related to TUG scores (*r* = −0.81) [21]. Furthermore, good discriminative ability of the Berg balance test was indicated in predicting falls in a community-based prospective study [22].

The main aim of this study was to investigate the types and co-occurrence patterns of physical frailty, poor cognitive function and low functional independence in a cohort of elderly participants, using a data-driven approach to derive clusters of the common phenotypes of ageing. Detecting such shared patterns of age-related decline and healthy ageing may provide a useful guide for further development of assessment criteria and test selection, and help to shape intervention and plans to support healthy ageing. Finally, most frailty research has been conducted in Western countries, including Canada, the USA, Europe and Australia [1,23,24,25,26,27,28,29]. The present study investigated a cohort based in Shanghai (China), adding to frailty research in Asia where very few similar studies having been conducted [30].

## 2. Methods

### 2.1. Participants

All 50 to 95-year old persons born between 1 May 1917 and 31 July 1962 who were registered for census purposes in the Chang Ning district of Shanghai, China were invited to take part in the study. From this initial pool (*n* = 367) a sample of 170 participants volunteered to participate. No information was available on non-responders. No monetary incentive was offered, but participants at the community health center were provided with a free lunch. Ethical approval was obtained from Shanghai Mental health center of Jiao Tong University, Shanghai, China (reference IRB00002733201219) and Loughborough University, Loughborough, UK (reference R12-P16).

### 2.2. Procedure

#### Translation and Testing Procedure

To ensure that the correct meaning of words was delivered during translating questions from English to the local language (Mandarin), multiple forward and backward translations were performed and the test pack was proof-read by members of the scientific staff in both China and Britain. Back translation was successful for all tests and questionnaires.

Information about the study was distributed to potential participants aged 50–95 via local community health centers. In collaboration with Shanghai Jiao Tong University, 5 clinicians, 7 research assistants and one experienced Field Coordinator were trained in administering and scoring the relevant tests. Training included pilot testing and feedback. Interested participants were asked to bring their caregiver (if they had somebody) and were tested between 8 and 11 a.m. at local health centers to avoid possible confounding effects of time of day.

### 2.3. Questionnaires and Tests

Demographics (e.g., age, gender, education), and other variables, such as health and lifestyle were assessed using standardized questionnaires. Cognitive and physical status was assessed thereafter. Physiological symptoms were measured by assessment of muscle strength (grip strength, the Timed Up and Go (TUG) Test, gait speed (15 feet walking test), balance (Berg Balance scale), and body mass index. Activities of daily living (ADL) were assessed through the ADL questionnaires. Cognitive capacity tests included the Mini Mental State Examination (MMSE) and the Hopkins verbal learning test (HVLT). Each test session took roughly 100 min in total and included two short breaks of 10 min.

### 2.4. Cognitive Assessments

#### 2.4.1. Mini Mental Status Examination (MMSE)

The Mini Mental Status Examination (MMSE) [4] is commonly used to measure global cognitive function. It assesses 5 cognitive domains: orientation, registration (immediate memory), short-term memory, attention and calculation and language.

#### 2.4.2. Hopkins Verbal Learning Test (HVLT)

The HVLT [31] is widely used to detect memory decline. It is a word learning test consisting of 12 words from 3 categories. Only version A was used in this study. Immediate recall (IR) was repeated 3 times for the total IR score. Delayed recall (DR) scores were obtained after a 20 min delay without repeating the words.

#### 2.4.3. Functional Ability Measures

Functional ability was measured using the Barthel Activity of Daily Living scale (ADL) [32]. The ADL was developed to examine participant’s basic functional status. It tests ten items including the ability to independently feed oneself, bathe, groom oneself, control of bowels and bladder, toilet use, transfers, mobility on level surfaces and stairs. A higher score on the 10 point scale for each question indicates more functional independence.

### 2.5. Physiology Assessments

#### 2.5.1. Grip Strength

Lower grip strength is associated with incident as well as prevalent disability and can be predictive of morbidity and mortality [17]. It was assessed using a digital hand dynamometer as the best results of 3 trials.

#### 2.5.2. Gait Speed—15 Feet Walking Test

Gait Speed is widely used as a standard in rehabilitation reflecting muscle strength and is usually assessed by a 15 feet walking test. This assesses how long it takes for a participant to walk at his/her own pace for a distance of 15 feet. Gait scores were ranked (faster scores received higher ranks) for further analysis to allow for the inclusion of participants unable to walk unassisted.

#### 2.5.3. Lower Body Strength—Timed-Up-and-Go (TUG) Test

The TUG score is measured as the time that a participant takes to rise from a chair, walk three metres, turn around, walk back to the chair and sit down. Scores reported here are based on the average of 3 test trials. Participant’s ability to stand up without physical assistance (*i.e.*, touching the chair armrest) was recorded subsequently. TUG scores were also ranked with highest ranks given for fastest times to allow for inclusion of all participants.

#### 2.5.4. Postural Stability—Berg Body Balance Test

Falls and fall-related injuries are a major health problem for the elderly. Balance is critical for the normal performance of physical activities, and impaired balance is an important risk factor for falls in older people. The 14 items being tested in the Berg balance test include sitting to standing, standing unsupported, standing to sitting, sitting unsupported, transfers, standing unsupported with eyes closed, standing unsupported with feet together, reaching forward with arms stretched while standing, reaching forward to place a ring around a standing stick, picking up objects from floor, looking behind while standing with feet fixed, turning 360 degrees, alternating placing foot on step, tandem stance, and standing on one leg [20]. On each task, scores range from 0 to 4, and a higher score represents better performance.

### 2.6. Statistical Analyses

Descriptive analyses were performed followed by partial correlation analyses corrected for age and education. Principal Component Analysis (PCA) followed by cluster analysis was used to extract common components of the key variables and clustering of the participants. All analyses were performed in R version 3.0.1 and the “cluster” package was used for cluster analyses. The significance cut-off was set at α = 0.05.

## 3. Results

### 3.1. Demographics and Cognitive Test Results

A total of 170 participants volunteered to participate. Due to missing data on 22 participants the final sample analyzed below included 148 participants. Key demographic characteristics are outlined in Table 1, including the mean age of 74 years (range 58–92) and the mean education level of about seven years with considerable variance (SD = 4.7). 23% of the current sample had a history of smoking and 18% reported previous alcohol use. The mean MMSE score of 24.5 was well above the dementia threshold, as was the HVLT total recall score of 17.3. The demographics and cognitive test results were similar to those reported in a larger community-based study from the same area in Shanghai, China (Xu, 2015) [14].

### 3.2. Correlations

A correlation analysis of 11 variables was first conducted to examine factorability including age, years of education, grip strength, Timed Up & Go (TUG) scores, 15 feet gait scores, Mini Mental Status Exam (MMSE) score, Hopkins Verbal Learning test (HVLT) immediate and delayed recall (IR and DR, respectively), Activities of Daily Living (ADL) score, Berg balance and body mass index (BMI). Due to poor correlation with the other variables BMI was removed; all other variables were significantly correlated. Age and education were significantly correlated with each other (*r* = −0.38, *p* < 0.001), and with all the outcome variables (*p* ≤ 0.002 in all cases). Given the high degree of correlations it was important to determine which cognitive and physical functions were particularly driven by age and/or education, and which of these were key predictors of the functionally important ADL.

To remove these underlying demographic effects, partial Pearson’s r correlations corrected for age and education with all the physical and cognitive variables are shown in Table 2. Education-corrected age was significantly correlated with all outcome variables, with especially strong correlations with gait and MMSE scores (*r* > 0.5 for both). Age-corrected education was a particularly strong predictor of the MMSE score (*r* = 0.6), but also of the HVLT delayed recall (*r* = 0.44) and 15 feet gait scores (*r* = 0.4). Finally, the key predictors of age and education-corrected ADL were balance (*r* = 0.54) and MMSE (*r* = 0.41), but both gait scores were also significantly correlated (*r* = 0.25) with ADL scores.

The age and education-corrected correlation coefficients between the physical and cognitive variables provide an overview of the key relationships between these domains (Table 3). Cognitive test were internally strongly correlated, with *r* ranging from 0.43 to 0.44 for MMSE and HVLT IR and DR, respectively. As expected, HVLT IR and DR scores were highly correlated at *r* = 0.60. The physical ability scores were similarly internally correlated, with *r* = 0.29 and 0.43 between grip strength and TUG gait and 15 feet gait scores, respectively. Berg balance scores were also significantly correlated with gait scores (*r* = 0.49 and 0.52 for TUG and 15 feet gait, respectively). The two gait scores were unsurprisingly highly correlated at *r* = 0.76. However, despite the significant balance—gait and grip strength—gait relationships, there was no significant correlation between grip strength and balance. Finally, no significant correlations between any of the cognitive and any of the physical variables remained after correcting for age and education.

### 3.3. Principal Component Analysis

The sample size of 148 was deemed to be adequate for a PCA with 10 variables as it left data 15 rows per variable. The PCA indicated that 74% of the variance was contained within the first three principal components (PCs), which were analyzed further.

The rotation matrix results detailing the factor loadings onto the first three PCs is listed in Table 4. If a cut-off score of 0.3 is assumed to represent a significant factor in each component, the first component included age (negative), all the cognitive scores (MMSE, and HVLT IR and DR), and the gait scores. This component explained 52% of the variance and appeared to represent age-related cognitive and physical decline, where education also plays a role, probably largely because of its correlation with age in this cohort, but also because it is a protective factor.

The second component, which explained 13% of the variance, was driven mainly by education, balance and the verbal memory scores HVLT IR and DR, with age being a relatively unimportant factor (−0.1 weight). This component hence appears to reflect largely education levels and cognitive decline, with the strong loading of balance probably reflecting neurological conditions that impact on balance and memory, rather than physical frailty *per se*. The third component with 8% of the variance was most strongly related to ADL scores, grip strength and balance, reflecting the importance of both of the latter functions for ADL.

**Figure 1:**
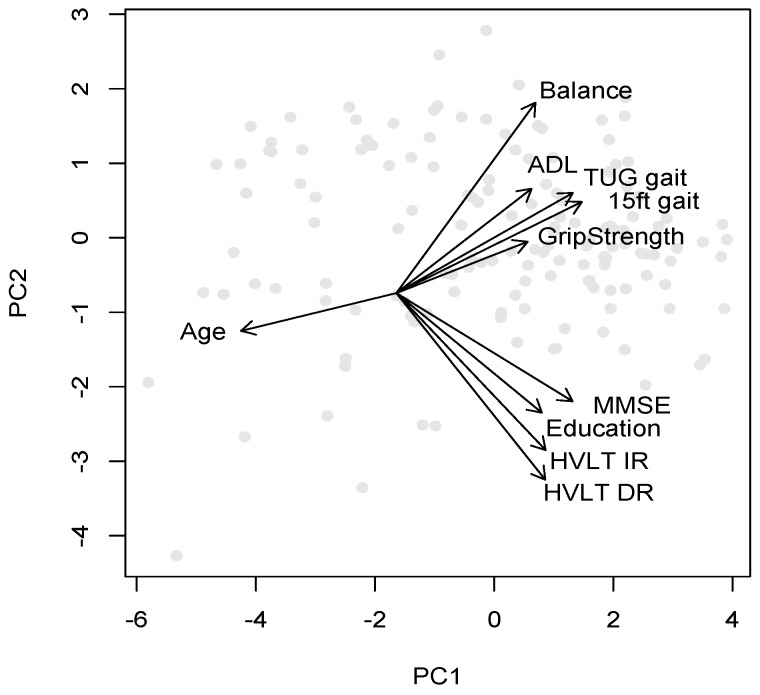
Mapping of the variables and participants (grey circles) onto the first 2 principal components.

The mapping of variables and participants onto the first two principal components is graphically illustrated in Figure 1, where the origin of the variable direction arrows has been shifted to optimize readability of the variable names. This mapping forms the basis for the cluster plots in the following section, where participants and clusters are plotted along the first two components.

### 3.4. Cluster Analysis

**Figure 2:**
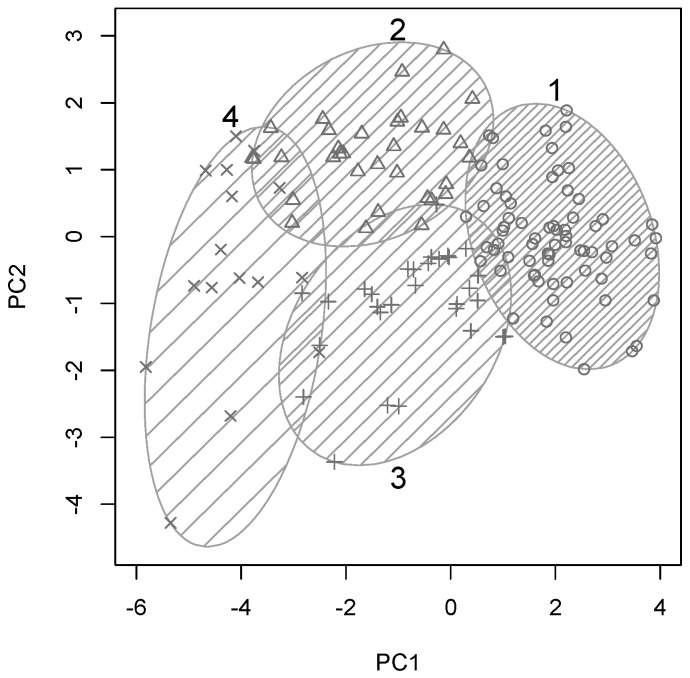
*K*-means clustering of all participants into 4 clusters, plotted along the first 2 components. Each cluster is numbered and shown as shaded ellipse. Shading density reflects within-cluster participant density and plotting symbols indicate cluster membership.

Ward’s minimum variance method was used to arrive at a hierarchical clustering structure for all participants based on their similarity across the 10 variables included in these analyses. A four-cluster solution yielded optimal cluster sizes for a systematic yet detailed group analyses, hence *k*-means clustering was used to separate all participants into four optimal clusters. This clustering solution is plotted along the first two principal components in Figure 2, with clusters shown by numbered ellipses and each participants plotted with one of four symbols to indicate cluster membership.

Cluster 1 (69/148 participants, 47%) appeared to contain the cognitively and physically healthy and mostly younger individual, as it falls on the “young end” of the PC1. At the other end of the spectrum, this cluster reflected largely age-related cognitive and physical decline as described above. Cluster 4 (16/148 participants, 11%) represented the opposite end of the spectrum and included the oldest participants with significant cognitive and physical impairments as it occupies the other end of PC1. Clusters 2 (33/148 participants, 22%) and 3 (30/148 participants, 20%) were situated in between these two extremes on PC1 and differed only with respect to their position along PC2, which was dominated by education and cognitive scores.

**Figure 3:**
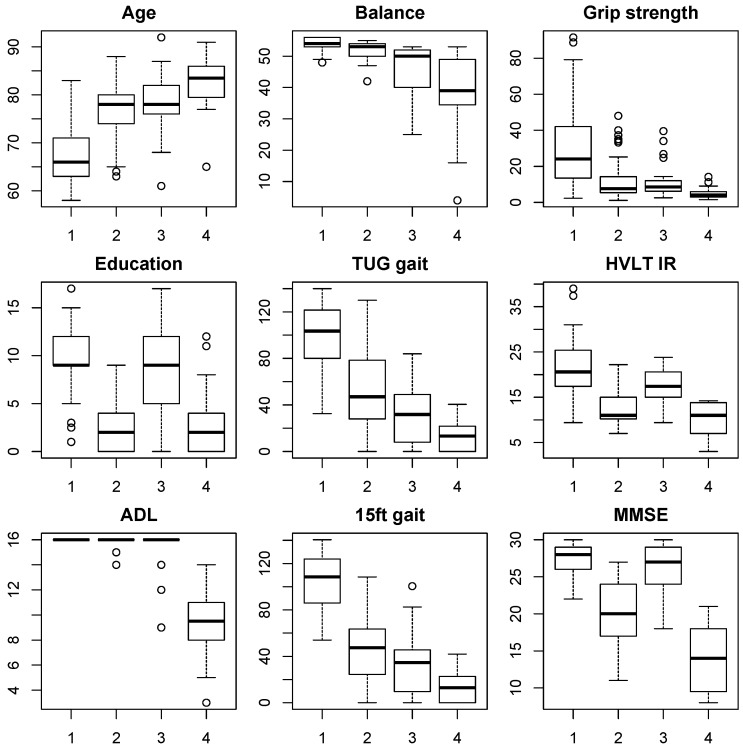
Box plots of the scores on the variables entered into the cluster analysis, split by cluster as indicated by the numbers on the *x* axis. HVLT DR scores are not included as the pattern is almost identical to the IR scores.

Hence, participants in clusters 2 and 3 were likely to be of similar age but with differences in education, balance and cognitive function. To investigate the characteristics of these four participant clusters in more detail, box plots of all original input variables for each of the four clusters are shown in Figure 3.

Figure 3 of the individual outcome measures in each cluster confirmed the indications derived from the positioning along PCs 1 and 2, as described above. The majority (47%) of elderly from this Shanghai census based cohort fell into group 1, which was young-old (median age 65 years), had good levels of education (median score 9) and was physically and cognitively robust with no impairments in ADL. In contrast, group 4 (11%) contained the oldest participants (median age 83 years), had low education levels (median 2.5 years), poor ADL, balance, gait, and grip strength scores, indicating the presence of frailty. Cognitive function scores were also very low with a median MMSE score of 14 and HVLT IR score of 11, indicating the presence of dementia.

The intermediate groups 2 and 3 (22% and 20%, respectively) each had the same median age of 78 years but differed significantly with respect to education levels, with a median education score of 2.5 in cluster 2 and a median education score of 9 in cluster 3, which was directly comparable to groups 4 and 1, respectively. Despite the similar ages in cluster 2 and 3, the most apparent difference was found in cognitive function, with the median HVLT IR score of 11 and median MMSE score of 19.5 for group 2 (low education) suggesting prevalence of dementia. In contrast, cognitive function in cluster 3 (high education) remained quite robust with a median MMSE score of 27 being directly comparable to the healthy younger cluster 1 and well above the borderline cut-off for even mild cognitive impairment (24.5). Nevertheless, some early signs of cognitive decline were apparent in verbal memory function with a median HVLT IR score of 17, which was lower than the healthy cluster 1 with a score of 21.

In contrast to cognitive function, physical function scores were actually slightly better in cluster 2 than in cluster 3. Median grip strength scores are almost identical, but both gait scores medians were slightly higher in cluster 2, as were balance scores. These differences were not significant, but they illustrate the fact that the protective effect of education on cognitive functions did not extend to physical outcomes. Furthermore, there were only 2 individuals with small reductions in ADL in group 2, hence the cognitive impairment did not seem to result in significant problems with ADL in this group. Similar good preservation of ADL was found for cluster 3, indicating that overall only the oldest cluster 4 with significant impairments in both physical and cognitive function experienced significant ADL reductions in this cohort.

## 4. Discussion

In this study we attempted to further investigate the relationship between cognitive and physical function in older age, and their respective impacts on ADL. Partial correlation analysis revealed that age was the main predictor of physical abilities, whereas education was the strongest predictor of cognitive functions. After correcting for age and education effects, independence in ADL was strongly related to good MMSE scores, confirming the functional relevance of this widely used test of global cognitive function. Balance scores were the strongest predictor, and the gait measures were also significantly correlated with ADL scores, underlining the importance of good mobility and balance for functional independence in old age. Surprisingly, grip strength was not correlated with ADL after correcting for age and education. However, it was low in all three older clusters (2–4), and significantly correlated with age and education, as well as the gait scores. In a community-based study conducted in the UK [11], grip strength proved to be a strongly predictive marker of frailty. In our study it was associated with gait, but not directly with reductions in ADL.

The relationship between cognitive and physical function revealed interesting patterns. First, there were significant correlations between all cognitive and physical outcome measures, which became non-significant after correcting for age and education. This implies that global cognitive function and verbal memory constitute an independent domain from physical mobility, balance and grip strength. Cognitive and physical function thus only showed related patterns of impairment because of the underlying shared determinants age and education. Hence it is important to assess memory, wider cognitive function, mobility and balance especially in older patients who are just beginning to develop signs of functional decline, as the pattern of affected domains can be quite heterogeneous.

Several studies reported that cognitive impairment was associated with physical frailty ([6,26,33,34]. Furthermore, frail persons were found to be at high risk of developing dementia, and hence poor physical function can assist in predicting dementia [26]. However, cognitive impairment was still the strongest predictor of dementia in this study because being frail with cognitive impairment increased the risk of developing dementia by almost a factor 5, compared to being frail without cognitive impairment (OR = 4.98, 95% CI = 2.17–11.41 *vs.* OR = 0.74, 95% CI = 0.27–2.07) [33]. Accordingly, Kulmala (2013) suggested that cognitive impairment should be considered to be included in the definition of frailty [33], but results from the present cohort suggest that cognitive and physical impairment may be less related once age and education are taken into account.

In the present study, older people (>79 years of age) who performed poorly on the physical tests were also at risk of cognitive impairment, although there was large variation (cluster 4). This group of people had poor balance (Berg scale <50), impaired scores on the gait tests as well as cognitive scores in the dementia range. A large part of the age and education effects was driven by the strong contrast to the overall healthy cluster 1, where participants were younger (<70 years), had much higher levels of education, and showed no signs of any physical, cognitive or ADL impairment.

The interesting variation in ageing patterns was revealed in the clustering solution in between these two outer ends of the ageing spectrum in clusters 2 and 3, who were of a matched intermediate age (median 78 years). They were just at the cusp of showing impaired physical function, but cluster 3 had much higher education levels (median nine years *versus* 2.5 in cluster 2), and correspondingly showed cognitive function levels very similar to the cognitively healthy and much younger cluster 1. In contrast, cluster 2 with low education had the same median HVLT scores as the old and impaired cluster 4, as well as MMSE scores closer to that group than cluster 3, and about half scored below even conservative cut-offs scores (15) for dementia on the MMSE. This extends the significant body of evidence that high education levels can offer significant protection against cognitive decline [14], and that verbal memory tests are particularly sensitive to education effects [35]. People with low education may thus show accelerated cognitive decline with age, and this needs to be corrected for in analyses [14].

Nevertheless, after correcting for age there was a stronger partial correlation between education and MMSE scores (*r* = 0.6) compared to HVLT scores (*r* = 0.36 for IR and 0.44 for DR), suggesting that MMSE scores were more sensitive to education than HVLT scores in the present study. The lower HVLT but not MMSE scores in the cognitively more robust cluster 3 compared to the overall healthy cluster 1 may perhaps reflect differences in cognitive reserve that could have been obscured by ceiling effects on the MMSE. Such ceiling and floor effects on the HVLT may go some way towards explaining the stronger education correlation with MMSE scores, and these results should be tested in larger cohorts with a wider range of cultural and ethnic backgrounds.

A key finding was that the protective effect of education on cognition did not appear to extend to physical function, because the better educated cluster 3 actually displayed slightly lower balance and gait scores than the age-matched cluster 2, and grip strength was very similar. Nevertheless, significant positive correlations between education and grip strength and gait scores remained after correcting for age, suggesting that education may also benefit some physical functions in old age. Again, larger cohort studies, ideally with lower correlations between age and education to disambiguate both effects, should be used to further investigate the effects of education and lifestyle on physical function in older age. Like dementia, frailty should also be differentiated from the normal ageing process.

According to a review by Collard [36], the prevalence of frailty in community studies ranged from 4.0% [25] to 59.1% [28], showing substantial variation. This may be due to the varied definition of frailty and use of different age groups from different socioeconomic communities and ethnicities. Others reported that the frailty rate increased gradually with an advanced age, from 4% in the 65–69 age group, to 26% in the 85 and above age group [37], which is similar to our findings. As defined by people needing help with ADL, our data suggest a 14% prevalence of frailty in the entire cohort (over 50 years of age), and 50% in those over 85 years of age.

However, this percentage is higher compared to the only existing Asian community-based study in which a frailty rate of 4.9% was reported using Fried Frailty Index (FFI) [30]. This rate is also higher than the figure from Fried’s study (6.9%) [1], but significantly smaller than another study in European using the FFI [27]. However, the pre-frailty rate in these three studies is similar to our results (40%, 46.6% and 43.5%, respectively).

The reason for this wide variation in prevalence rates is not entirely clear, e.g., whether there are ethnic differences in frailty phenotypes or the applicability of the frailty diagnostics. The Chinese cohort described here comprised elderly volunteers who survived a childhood without antibiotics and experienced periods of famine and war, which may have led to a healthy survivor and volunteer bias. Subsequent analyses should use a similar approach in cohorts of different ethnic and geographical backgrounds to investigate whether the effects found here apply to elderly people across other cultures and locations. However, prevention by identifying modifiable risk factors is likely to work best when taking regional and cultural specifics into account. For instance, Woo (2002) reported that with increasing urbanization in China in the past decades, levels of physical activity are reduced [38]. There is also a rural-urban discrepancy in nutritional intake (e.g., 12%–18% of energy is derived from fat in rural areas *versus* 20%–31% in urban areas) further exacerbating the risk for obesity and related morbidity (diabetes, heart disease, dementia). Poor nutrition and lack of activity have been associated with dementia and frailty [1], which might suggest that frailty and cognitive impairment rates may be on the rise in Shanghai, but these factors may not apply in other locations or countries.

Another limitation of the current study is the relatively small sample size. A three-cluster solution was very stable, which essentially grouped the young and healthy separate from an intermediate (by age and cognitive/physical scores) group that included clusters 2 and 3 as described above, and an older and more impaired group similar to cluster 4. However, five or six-cluster solutions would also be possible based on statistics, but then the group size in each cluster became very small so effects became unstable and fragmented. The four-cluster solution presented here was chosen for optimal balance of detail in the group split whilst also preserving reasonable cluster sizes that allowed for a good theoretical interpretation. Larger cohort analysis with better power could investigate more refined cluster solutions to detect more detailed ageing sub-types.

Overall, the majority of participants in the present cohort were cognitively and physically healthy and reported no problems with ADL. Even though this included mostly the younger participants, it is nevertheless an encouraging sign of active and healthy ageing in this cohort. In sum, 28% of participants needed help with ADL, with the majority of this group being over 80 years of age. Significant predictors of reductions in functional independence included age, balance, global cognitive function (MMSE) and the gait measures. Cluster analysis revealed a protective effect of education on cognitive function which did not appear to extend to physical function, although several of the physical function outcome measures did show partial correlations with education after correcting for age. Evidence-based models for dementia and frailty prevention measures may be derived if the ageing clusters found here prove to be consistent in other ageing cohort studies.

## 5. Conclusions

We can conclude that using cluster analyses in this older cohort from Shanghai, 4 different types of participants were identified:

i) most (47%) who were healthy and on average slightly younger (75 years on average, range 58–85) with good obtained education (10 years average);
ii) 22% who had mainly cognitive problems (dementia) but were otherwise fit (22%, low education, 79 years of age, range 65–88);
iii) those who had cognitive function above the dementia thresholds but had poor gait, balance and grip strength (20% high education, 79 years of age, range 70–87); and
iv) 11% the very old who were physically frail and had dementia with high care needs (low education, 85 years on average range 78 to 92 years).

This reflects other data showing that education can protect against dementia but not physical frailty. These clusters can inform designers, policy makers and treatment studies focused on lifestyles as not all old people have the same needs and cognitive/physical abilities. We are currently carrying out similar analyses in UK cohorts to confirm these clusters.

## Figures and Tables

**Table 1:** Key demographics and cognitive test results.

Variable	Mean (SD/Range/Ratio)
*N*	148
Age	73.5 (58–92)
Education (years)	7.1 (4.7)
Gender (female)	103 (60.6%)
Smoking history	39 (22.9%)
Alcohol use history	31 (18.2%)
MMSE total score	24.5 (5.4)
HVLT total recall	17.3 (6.2)

**Table 2:** Partial correlations of all variables with age (corrected for education), education (corrected for age) and ADL (corrected for age and education). Only Pearson’s *r* values significant at *p* < 0.05 with a Bonferroni-Holm correction for all comparisons are shown. Abbreviations: ADL—activities of daily living, MMSE—Mini Mental Status Exam, HVLT—Hopkins Verbal Learning Test (IR—Immediate Recall, DR—Delayed Recall), TUG—Timed Up & Go.

Variable	Age	Education	ADL
ADL	−0.32	-	-
Grip Strength	−0.30	0.23	-
Balance	−0.36	-	0.54
TUG gait	−0.53	0.28	0.25
Gait 15 feet	−0.55	0.40	0.25
MMSE	−0.38	0.60	0.41
HVLT IR	−0.29	0.36	-
HVLT DR	−0.30	0.44	-

**Table 3:** Partial correlation matrix between the physical and cognitive variables, controlled for age and education. Only Pearson’s *r* values significant at *p* < 0.05 with a Bonferroni-Holm correction are shown. Abbreviations: ADL—activities of daily living, MMSE—Mini Mental Status Exam, —Hopkins Verbal Learning Test (IR—Immediate Recall, DR—Delayed Recall), TUG—Timed Up & Go.

Variable	Balance	TUG Gait	Gait 15 Feet	MMSE	HVLT IR	HVLT DR
Grip Strength	-	0.29	0.43	-	-	-
Balance	-	0.49	0.52	-	-	-
TUG gait	0.49	-	0.76	-	-	-
Gait 15 feet	0.52	0.76	-	-	-	-
MMSE	-	-	-	-	0.43	0.44
HVLT IR	-	-	-	0.44	-	0.60

**Table 4:** Variance explained and factor loadings for the first 3 components. Significant variables with a factor of at least 0.3 are highlighted in bold. Abbreviations: ADL—activities of daily living, MMSE—Mini Mental Status Exam, HVLT—Hopkins Verbal Learning Test (IR—Immediate Recall, DR—Delayed Recall), TUG—Timed Up & Go.

Variable	PC 1	PC 2	PC 3
Variance explained	52.4%	13.4%	8.4%
Age	**−0.32**	−0.10	−0.14
Education	**0.30**	**−0.30**	0.12
Grip strength	0.27	0.13	**0.56**
TUG gait	**0.36**	0.25	0.18
15 feet gait	**0.38**	0.23	0.26
Balance	0.28	**0.48**	**−0.30**
ADL	0.28	0.26	**−0.63**
MMSE	**0.36**	−0.27	−0.13
HVLT IR	**0.30**	**−0.39**	−0.18
HVLT DR	**0.30**	**−0.47**	−0.10
